# Where Does Liquid Biopsy Add Value in Thyroid Cancer? Biological Rationale, Technological Innovation, and Clinical Utility

**DOI:** 10.3390/biomedicines14061274

**Published:** 2026-06-02

**Authors:** María Alonso-Chamorro, Ainhoa Palacios Mejorada, Garcilaso Riesco-Eizaguirre

**Affiliations:** 1 Molecular Endocrinology Group, Faculty of Medicine, Universidad Francisco de Vitoria, 28223 Madrid, Spain; m.alonso.prof@ufv.es (M.A.-C.); ainhoa.palacios@ufv.es (A.P.M.); 2 Department of Endocrinology & Nutrition, Hospital Universitario de Móstoles, 28935 Madrid, Spain

**Keywords:** liquid biopsy, ctDNA, CTCs, extracellular vesicles (EVs), microRNAs, thyroid cancers

## Abstract

Thyroid cancer comprises biologically diverse entities ranging from largely indolent differentiated thyroid cancer (DTC) to aggressive poorly differentiated/anaplastic thyroid cancer and medullary thyroid cancer, generating a need for minimally invasive biomarkers that can be repeatedly sampled. This review summarizes recent advances in liquid biopsy for thyroid cancer, focusing on analytes and technologies spanning circulating tumor DNA (ctDNA)/cell-free DNA, circulating microRNAs (miRNAs), extracellular vesicles (EVs), and circulating tumor cells (CTCs). For ctDNA, we contrast qPCR/ddPCR and next-generation sequencing, tumor-informed versus tumor-agnostic strategies, the impact of low tumor fraction in DTC, clonal hematopoiesis confounding, and emerging methylation-based multi-cancer detection paradigms. For miRNAs, we highlight that bulk serum/plasma and EV-enriched compartments are not interchangeable and that regulated EV loading supports fraction-resolved biomarker development. We review recent translational EV-miRNA studies, including externally validated classifiers for metastatic disease and follicular-patterned/indeterminate nodules, and summarize the evolution of CTC research from enumeration to preoperative risk stratification and postoperative or radioiodine-related kinetics. We conclude with an indications-first framework that pairs analyte choice with clinical intent (preoperative diagnosis, initial risk stratification, response to treatment and minimal residual disease and identification of actionable alterations and resistance mechanisms) and prioritizes standardized workflows and prospective multicenter validation. Multi-analyte integration, epigenetic/fragmentomic cfDNA signals, and higher-resolution EV analytics are likely to accelerate clinical adoption, particularly in advanced thyroid cancer where circulating signal and therapeutic actionability are highest.

## 1. Introduction

**Figure 1:**
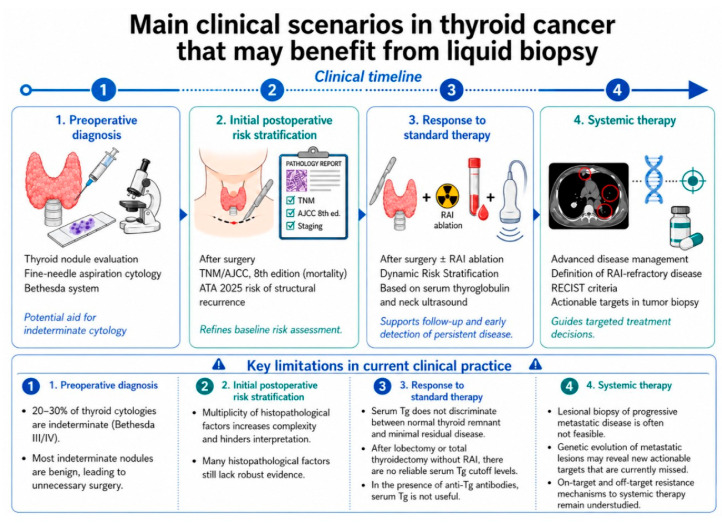
Integrated liquid biopsy framework in thyroid cancer, highlighting major clinical scenarios and current limitations across diagnosis, risk refinement, surveillance, and advanced disease management.

Thyroid cancer is the most prevalent endocrine malignancy and encompasses biologically diverse entities that range from typically indolent differentiated thyroid cancers (DTC; papillary (PTC) and follicular (FTC)) to aggressive poorly differentiated and anaplastic thyroid carcinoma (ATC), as well as medullary thyroid cancer (MTC) arising from parafollicular C cells [1]. In parallel with the global rise in detection—driven in part by imaging and surveillance—clinical practice has increasingly embraced risk-adapted and de-escalation paradigms in DTC, while intensifying molecularly guided systemic therapy in advanced, radioiodine-refractory (RAIR), and anaplastic disease [2]. Across this spectrum, a central unmet need persists: scalable biomarkers that (i) refine diagnosis in indeterminate nodules (notably follicular-patterned lesions), (ii) capture tumor aggressiveness beyond static histopathology, (iii) enable dynamic monitoring of disease burden and treatment response when conventional markers are equivocal or biologically constrained, and (iv) guide targeted systemic treatment decisions when lesional biopsy of progressive metastatic disease is not possible. Figure 1 summarizes the main clinical scenarios that may benefit from liquid biopsy and the key limitations in current clinical practice.

Liquid biopsy—broadly defined as the interrogation of tumor-derived material in biofluids—has emerged as a minimally invasive route to longitudinal molecular profiling [3]. Conceptually, liquid biopsy complements tissue genotyping by addressing spatial and temporal heterogeneity, enabling repeated sampling, and potentially anticipating radiographic progression through real-time molecular kinetics. In thyroid cancer, however, translation is intrinsically context dependent: many DTCs exhibit low tumor shedding into the circulation, which limits sensitivity for circulating tumor DNA (ctDNA) and circulating tumor cells (CTCs), whereas aggressive subtypes and higher-burden disease provide more favorable conditions for detection and monitoring [4,5]. The most recent thyroid cancer guidelines still recommend surgical or core tumor biopsy and re-biopsy over liquid biopsy, which may be considered (particularly ctDNA) for patients with advanced thyroid cancer in whom tumor biopsy is not possible [2,6]. Consequently, the field is shifting from proof-of-detectability toward a more clinically disciplined question: in which thyroid cancer scenario does a given liquid biopsy analyte change management?

**Figure 2:**
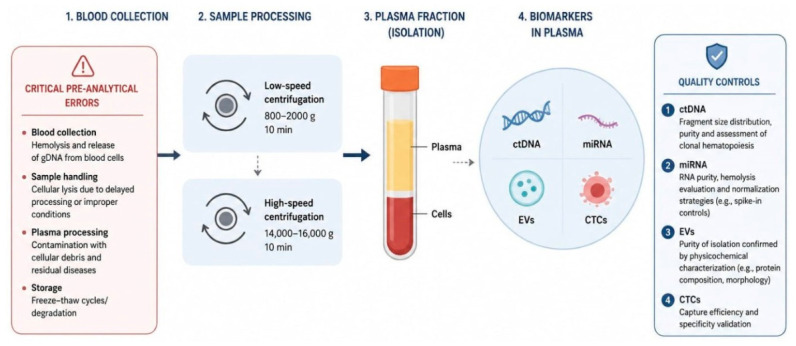
Pre-analytical and analytical considerations in liquid biopsy workflows. The liquid biopsy process involves (1) peripheral blood collection; (2) sequential centrifugation to separate plasma from cellular components; (3) isolation of the plasma fraction; and (4) analysis of key circulating biomarkers, including circulating tumor DNA (ctDNA), microRNAs (miRNA), extracellular vesicles (EVs), and circulating tumor cells (CTCs). Pre-analytical variables at each step may introduce critical errors that affect biomarker yield and integrity. Appropriate quality controls are essential to ensure the reliability and reproducibility of liquid biopsy analyses.

It is important to note that techniques for the isolation and detection of liquid biopsy analytes are continuously evolving; however, the lack of standardized protocols remains a major limitation, hindering reproducibility and cross-study comparability. While certain pre-analytical variables are shared across analytes, each biomarker requires specific and well-defined quality control measures to ensure analytical reliability. Figure 2 illustrates the critical pre-analytical steps in liquid biopsy that are shared across analytes, together with the minimum analyte specific quality control requirements necessary to ensure standardization and reproducibility.

This review synthesizes recent advances in thyroid cancer liquid biopsy with an explicit emphasis on how technological innovations are enabling clinically meaningful applications—from diagnosis, risk refinement and minimal residual disease (MRD) concepts to therapy selection, response tracking, and emerging therapeutic interventions. The manuscript is structured by analyte class, reflecting both biological rationale and technological advances. First, we cover circulating tumor nucleic acids, focusing on ctDNA mutation detection (qPCR/ddPCR vs. NGS panels), tumor-informed versus tumor-agnostic strategies, key confounders such as clonal hematopoiesis, and the expanding role of cfDNA methylation and multi-cancer detection paradigms—while critically appraising why sensitivity remains stage and histotype dependent in thyroid cancer. Second, we address circulating miRNAs, highlighting the clinically consequential distinction between “bulk” serum/plasma miRNAs and vesicle-enriched miRNAs, and why compartment selection (and workflow harmonization) can determine reproducibility and clinical validity. Third, we examine extracellular vesicles (EVs) as a biomarker substrate with enhanced biological stability and regulated cargo loading, summarizing the evolution from exploratory signatures to externally validated EV-miRNA classifiers that target concrete clinical gaps such as RAIR identification, metastatic risk, and follicular carcinoma diagnostics. Finally, we review CTCs, emphasizing technological progress in capture/detection (including receptor-based and nanoparticle-enabled platforms) and the emerging shift from enumeration to phenotype-informed and decision-relevant applications (e.g., preoperative risk enrichment, postoperative surveillance, and pharmacodynamic monitoring).

## 2. Searching Strategy

A comprehensive literature search was conducted across PubMed/MEDLINE, Web of Science, and Scopus databases to identify relevant studies published up to January 2026. The search strategy employed a combination of Medical Subject Headings (MeSH) terms and free-text keywords, including ‘thyroid cancer’, ‘liquid biopsy’, ‘circulating tumor DNA’, ‘cell-free DNA’, ‘microRNA’, ‘extracellular vesicles’, ‘exosomes’, and ‘circulating tumor cells’. Inclusion criteria focused on original research articles, systematic reviews, and meta-analyses that evaluated the diagnostic, prognostic, or monitoring utility of liquid biopsy analytes in differentiated, medullary, and anaplastic thyroid carcinoma. Studies were prioritized based on their clinical relevance, sample size, and methodological rigor. Only peer-reviewed articles published in English were considered. The reference lists of retrieved articles were manually screened to identify additional relevant publications, ensuring a robust and balanced synthesis of the current state of the field.

## 3. Circulating Tumor Nucleic Acids

### 3.1. Circulating Tumor DNA

**Figure 3:**
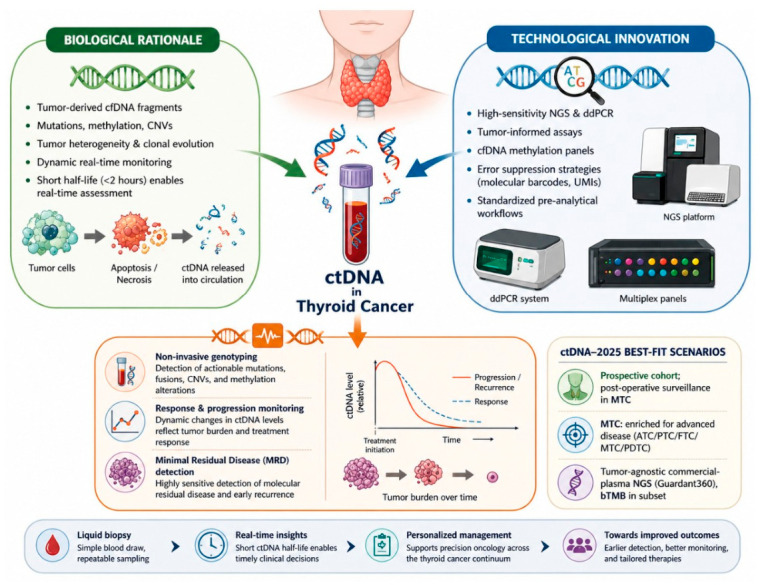
Integrated overview of circulating tumor DNA (ctDNA) in thyroid cancer, illustrating its biological basis, key technological advances enabling detection, and principal clinical applications across genotyping, disease monitoring, and minimal residual disease assessment.

Circulating tumor DNA (ctDNA) represents the fraction of circulating cell-free DNA (cfDNA) that originates from tumor cells and is released into body fluids—primarily peripheral blood—as short fragments derived from apoptosis, necrosis, and active secretion, carrying somatic alterations that mirror the tumor genome (see Figure 3). This “tumor-derived” nature theoretically allows a simple blood draw to function as a repeatable sampling of the cancer molecular landscape: ctDNA can capture both inter- and intratumoral heterogeneity, identifying actionable targets, and, owing to its short half-life (reported to be <2 h), enables near-real-time molecular monitoring of tumor dynamics and clonal evolution under therapy [7,8]. From a clinical standpoint, this temporal plasticity translates into three main axes of potential utility: non-invasive genotyping to identify actionable targets, treatment monitoring (including early detection of progression), and detection of minimal or molecular residual disease (MRD) in settings of low tumor burden where conventional techniques are delayed or inconclusive [8,9,10].

The principal structural challenge of ctDNA—particularly pronounced in thyroid cancer—is that it circulates as a minor fraction within total cfDNA, requiring highly sensitive analytical methods with stringent error control at both preanalytical and bioinformatic levels [4]. Moreover, ctDNA detectability varies according to disease severity, tumor burden, anatomical distribution, and tumor biology; in a landmark pan-cancer cross-sectional study, ctDNA was detectable in >75% of several advanced malignancies, whereas in others, ctDNA was detectable in fewer than 50% of cases, including thyroid cancer, anticipating limited baseline sensitivity in localized or indolent disease [11]. Methodologically, the literature converges on two major strategies: (i) selective amplification approaches (qPCR and ddPCR), offering high analytical sensitivity for known mutations but limited multiplexing, and (ii) sequencing-based approaches, which expand genomic coverage at the expense of increased complexity, depth requirements, and interpretative burden [4,5]. Superimposed on this dichotomy is a third, increasingly influential axis: “tumor-informed” assays (designed from the molecular profile of the primary tumor) versus “tumor-agnostic” assays (broad commercial plasma panels), with direct implications for MRD detection—more favorable to tumor-informed designs—versus discovery of actionable alterations in the absence of tissue—favored by broad panels [12,13].

A major biological limitation of cfDNA analysis is that the vast majority of fragments present in plasma originate from hematopoietic cells. With aging, these cells can accumulate somatic mutations through clonal hematopoiesis (CH), which represents a significant source of false-positive findings in liquid biopsy analyses. If CH-associated variants are not accurately distinguished from tumor-derived mutations, there is a risk of misinterpreting residual disease after tumor resection or inferring an inadequate response to therapy. Such misclassification may ultimately lead to inappropriate clinical decision-making and suboptimal patient management.

Highly sensitive assays enable the detection of CH mutations present in very small clonal populations. The limit of detection of an NGS assay is directly dependent on sequencing depth, meaning that variants with very low variant allele frequencies (VAF), including CH-associated mutations below 0.5%, can be reliably identified with ultra-deep sequencing. These observations underscore the importance of incorporating matched white blood cell (WBC) sequencing into cfDNA analysis pipelines, even in tumor-informed approaches, to confidently exclude CH-derived variants. Notably, the clinical implications of CH are not restricted to liquid biopsy, as similar confounding effects may arise during tumor tissue profiling potentially leading to the erroneous identification of targetable alterations. The current approach is performing paired sequencing of plasma cfDNA and DNA from WBC. However, the inherent biological mosaicism of hematopoiesis continues to challenge the specificity of circulating tumor DNA (ctDNA) detection. The additional cost and complexity associated with this approach may limit its widespread implementation in routine clinical practice [14,15].

Building on these ctDNA-based principles, multi-cancer detection (MCD) assays have recently emerged as an extension of cfDNA analysis, leveraging genome-wide methylation signatures to detect and localize signals from multiple malignancies through a single blood test [16,17,18]. From a thyroid cancer perspective, MCD testing illustrates a central paradox: although thyroid cancer–associated signals are detectable and standardized follow-up algorithms typically recommend thyroid ultrasound, the added clinical value is uncertain given the high prevalence of indolent disease, incidental nodules, and the absence of evidence that population-level molecular screening reduces thyroid cancer–specific mortality. Consequently, current evidence positions MCD assays as complementary exploratory tools rather than replacements for established screening strategies, with thyroid cancer representing a paradigm case where biological detectability may outpace demonstrable clinical benefit.

A recent narrative synthesis in thyroid cancer underscores the contrast between a strong biological rationale and persistent analytical limitations, emphasizing that ctDNA can encode single-nucleotide variants, methylation changes, and copy-number alterations, yet its typically low abundance—particularly in differentiated tumors without bulky disease—constrains sensitivity in real-world settings [4]. In PTC, plasma detection of BRAF alterations is consistently enriched in patients with higher tumor burden, invasive features, or distant metastasis, but results remain heterogeneous across studies; for example, one cited series reported BRAF V600E ctDNA positivity in 44.07% with 61.54% sensitivity and 90.91% specificity for malignancy discrimination in the studied context, whereas other cohorts failed to detect BRAF even using sensitive approaches, underscoring strong stage- and workflow-dependence [4]. The same review highlights emerging epigenetic signals—e.g., cfDNA hypermethylation involving MGMT and iodide-handling genes such as SLC5A8/SLC26A4—with one cited study reporting positive methylation indices in 70% of patients with conventionally confirmed recurrence, suggesting complementary value for relapse surveillance when standard markers are imperfect [4]. A systematic review and meta-analysis quantifies this variability across the literature to September 2022: among 36 included studies (*n* = 2566), 19 reported mutated ctDNA in thyroid cancer, and pooled diagnostic performance for plasma BRAF V600E in PTC (11 studies) showed moderate sensitivity (56%, 95% CI 36–74) but high specificity (91%, 95% CI 84–95), with an overall diagnostic odds ratio ~12—characteristic of a “rule-in” rather than “rule-out” test—while reiterating that robust clinical translation will require harmonized preanalytics/analytics, subtype- and stage-stratified cohorts, and standardized reporting thresholds [5].

Moving from systematic reviews to recent clinical studies (Table 1), a perioperative cohort study evaluated ctDNA before and after surgery in sporadic MTC (*n* = 29), excluding germline RET mutations, with staging-based stratification and a second postoperative sampling at a median of 8 months [12]. The design incorporated rigorous preanalytical handling (EDTA blood, plasma separation within <1 h, double centrifugation, extraction from 4 mL plasma) and a customized thyroid NGS panel covering the full coding regions of RET and RAS (H/K/N) and hotspot regions of 13 additional genes, with deep sequencing (~2000× in tissue and ~20,000× in ctDNA) and a positivity threshold defined as variant allele frequency (VAF) > 0.4% [12]. The findings were clinically paradoxical yet informative: preoperative ctDNA positivity was low (4/26; 15.4%), limiting diagnostic value in intrathyroidal disease, but was associated with RET M918T mutations, higher tumor VAF, and increased rates of persistent disease on follow-up; postoperatively, 3/23 (13%) patients were ctDNA-positive and showed higher calcitonin and CEA levels, suggesting added prognostic and monitoring value in patients with more aggressive biology or higher tumor burden. Explicit limitations included the relatively high VAF cutoff—raising the detection limit for low-level MRD—as well as small sample size and variability in postoperative sampling timing [12].

At a population scale, a retrospective database analysis evaluated tumor-agnostic plasma NGS in 1094 adult cases coded as thyroid cancer (samples collected 2016–2021), using a commercial plasma panel (Guardant360) and calculating blood tumor mutation burden (bTMB) in a subset [13]. This was the only study in our comparative analysis that explicitly accounted for clonal hematopoiesis, thereby reducing the risk of false-positive plasma findings. The cohort was dominated by “not otherwise specified” cases (876/1094), with histological subtype available in ~20% (92 anaplastic, 62 papillary, 14 follicular, 16 oncocytic, 2 poorly differentiated, 32 medullary), a median age of 65 years, and 47.3% male patients, consistent with advanced disease requiring systemic genotyping [13]. Clinical utility emerged from the actionable alteration landscape: 78.3% harbored ≥1 detectable alteration; TP53 was the most frequent overall; BRAF V600E was detected in 27.2% of anaplastic and 35.7% of papillary cancers (0% in follicular), while RAS mutations predominated in follicular carcinoma (62.5%); gene fusions (RET/ALK/NTRK) were rare but present, and RET mutations were identified in 66.7% of medullary cases. Mean bTMB was higher in anaplastic carcinoma compared with other subtypes, reinforcing the value of plasma-derived biomarkers for biological stratification [13]. The main limitation was conceptual and clinical: without systematic tissue comparison or detailed annotation of stage and treatments, this approach demonstrates feasibility and is hypothesis-generating but does not establish concordance or outcome impact—requirements for transforming a “genomic landscape” into a validated clinical biomarker.

In 2025, three studies illustrate methodological diversification, summarized in Table 1. First, a cross-sectional feasibility study applied a tumor-informed digital PCR approach to detect patient-specific alterations in both ctDNA and cfRNA (including fusion transcripts) in 34 patients sampled between 2021 and 2023, correlating results with ATA 2015 response categories [19]. Clinical performance was strongly disease-state dependent: ctDNA/ctRNA was detectable in all cases with structural incomplete response, with full concordance with imaging, whereas 91% of patients with an excellent response had undetectable ctDNA/ctRNA; indeterminate and biochemical incomplete responses showed variability, including one case with undetectable thyroglobulin but detectable ETV6::NTRK3 fusion in cfRNA, highlighting the added value of ctRNA integration for fusion detection when protein markers are inconclusive [19]. Second, in anaplastic thyroid carcinoma, a retrospective cohort study evaluated ctDNA as a surveillance biomarker: 45 patients with baseline sampling prior to systemic therapy (February 2021–August 2024) showed concordance between ctDNA status and disease state in 93%; during follow-up (31 patients; 130 results), sensitivity for detecting recurrence or progression was ~77–78%, with specificity and positive predictive value of 100% in both a post-curative-intent recurrence cohort and a residual-disease monitoring cohort. False negatives were attributed to low tumor burden and restricted metastatic patterns (lung/brain) or molecular heterogeneity [20]. Third, a prospective cohort of advanced/metastatic thyroid cancer (2020–2024; *n* = 40: 27 medullary, 11 poorly differentiated, 2 anaplastic) sequenced cfDNA collected in Streck tubes using a 50-gene panel on the Ion Torrent Genexus platform; mutations were detected in cfDNA in 18/36 (50%) patients with known tumor mutations, with markedly higher sensitivity prior to initiation of tyrosine kinase inhibitors (86%; 6/7) than during treatment (54%; 13/24), consistent with reduced ctDNA shedding under on-target response. Additionally, cfDNA concentration was higher in mutation-positive samples, and rising cfDNA levels were associated with worse progression-free survival [21]. This study underscores some limitations: small subtype-specific sample sizes and panel constraints (e.g., lack of TERT promoter coverage and limited fusion detection), which are particularly relevant alterations in aggressive thyroid cancer biology.

Collectively, recent advances suggest that ctDNA in thyroid cancer is transitioning from the question “can we detect it?” to “in which clinical scenario does it change management?”, with the answer appearing to be subtype and context dependent. Early differentiated thyroid cancer remains constrained by low ctDNA shedding, reflected in moderate sensitivities in BRAF V600E meta-analyses, whereas aggressive subtypes and advanced disease provide a window in which ctDNA/cfDNA dynamics may refine ATA 2025 response to treatment classification, anticipate progression and treatment response to systemic therapy or inform targeted therapies and treatment resistance, even when protein markers such as thyroglobulin, calcitonin, or CEA are ambiguous or pharmacologically modulated [5,19,22]. Two methodological gaps emerge as decisive for translating observations into clinically actionable biomarkers: first, standardization of preanalytical workflows and calling thresholds—as exemplified by the impact of relatively high VAF cutoffs in perioperative studies—and second, safeguarding specificity against clonal hematopoiesis through leukocyte controls or robust interpretative frameworks, to prevent biological “noise” from being misread as tumor progression or resistance [12,14].

### 3.2. Circulating Tumor miRNAs

Circulating microRNAs (miRNAs) are small non-coding RNAs, approximately 19–25 nucleotides in length, that regulate gene expression post-transcriptionally and have been extensively implicated in thyroid tumorigenesis, dedifferentiation, invasion, and metastatic progression. In liquid biopsy, miRNAs are attractive because they can be detected in serum or plasma using relatively accessible technologies, including quantitative reverse-transcription PCR (qRT-PCR), digital PCR, microarrays, and next-generation sequencing. However, circulating miRNAs should not be considered a single analytical entity. They may circulate as non-vesicular molecules bound to Argonaute proteins or lipoproteins, or they may be enclosed within extracellular vesicles (EVs). These compartments differ in biological origin, stability, pre-analytical requirements, and clinical interpretability. In this subsection, we focus primarily on free or bulk circulating serum/plasma miRNAs. EV-associated miRNAs, including their regulated loading, methodological requirements, and thyroid cancer-specific studies, are discussed in detail in the dedicated EV section below.

When working with circulating miRNAs, rigorous pre-analytical and analytical quality control is essential. RNA purity is commonly assessed by spectrophotometric absorbance ratios. An A260/A280 ratio of approximately 1.8–2.0 is generally considered compatible with adequate nucleic acid purity, whereas lower values may indicate protein contamination. Similarly, an A260/A230 ratio of approximately 2.0–2.2 supports acceptable RNA purity, while lower values may suggest contamination by phenol, salts, or other organic compounds used during extraction. In addition to RNA quantity and purity, circulating miRNA studies require specific controls. Exogenous spike-in controls are strongly recommended to monitor extraction efficiency, reverse transcription performance, and inter-sample technical variability. Assessment of hemolysis is also critical because erythrocyte-derived miRNAs can substantially distort circulating miRNA profiles; this is commonly evaluated using hemolysis-sensitive ratios such as miR-451/miR-23a.

The diagnostic potential of circulating serum/plasma miRNAs in thyroid cancer has been supported by systematic reviews, but translation remains limited by methodological heterogeneity. Xu et al. and Geropoulos et al. are among the most relevant recent systematic analyses dedicated to circulating miRNAs in thyroid cancer [23,24]. Xu et al. performed a diagnostic meta-analysis of serum/plasma RT-qPCR studies and reported overall moderate-to-good diagnostic performance, with pooled sensitivity and specificity around 0.81–0.85 and an AUC close to 0.88–0.89 [23]. Importantly, multi-miRNA panels outperformed single-miRNA assays, reaching an AUC of approximately 0.94, supporting the concept that combined signatures may better capture the molecular heterogeneity of thyroid nodules than isolated markers. Nevertheless, the analysis also emphasized substantial heterogeneity related to specimen type, patient selection, miRNA extraction protocols, normalization strategies, and assay platforms.

Geropoulos et al. provided a more recent and biologically nuanced synthesis, highlighting that most available evidence still derives from bulk serum or plasma, whereas a smaller subset of studies evaluates EV-associated miRNAs [24]. Within bulk serum/plasma studies, recurrent candidates include canonical PTC-associated miRNAs such as miR-146b, miR-221, and miR-222, as well as multi-miRNA panels including let-7e, miR-151-5p, and miR-222 [24]. These miRNAs are biologically plausible because they overlap with tissue-based thyroid cancer signatures and pathways involved in MAPK signaling, proliferation, invasion, and epithelial–mesenchymal transition. However, their diagnostic performance has varied across studies, indicating that bulk circulating miRNA measurements are sensitive to background biological noise and technical variability.

Two recent original studies illustrate both the promise and limitations of free circulating miRNAs. Verrienti et al. used a targeted serum approach and reported that selected canonical miRNAs, including miR-146a and miR-221, were associated with PTC diagnosis and features of progression [25]. These data suggest that, when analytically robust assays are applied to biologically prioritized candidates, free circulating miRNAs may provide clinically meaningful information. In contrast, Jankovic et al. used small-RNA sequencing and two independent qPCR platforms in a recurrence-focused DTC cohort, including longitudinal rhTSH stimulation, and failed to demonstrate consistent differences in known serum miRNAs between patients with recurrent/persistent disease and those in complete remission [26]. This study also highlighted platform discordance and small effect sizes, underscoring the difficulty of developing reproducible serum miRNA biomarkers for monitoring residual or recurrent disease.

Overall, bulk circulating miRNAs remain a promising but still immature class of liquid biopsy biomarkers in thyroid cancer. Their greatest potential currently lies in diagnostic support for thyroid nodules and PTC, particularly through multi-miRNA panels rather than single markers. However, clinical implementation requires harmonized pre-analytical workflows, standardized normalization strategies, careful hemolysis control, and prospective validation in independent multicenter cohorts. Importantly, future studies should clearly distinguish bulk serum/plasma miRNAs from EV-associated miRNAs, since the latter represents a biologically and analytically distinct compartment that may provide additional tumor-specific information, as discussed in the following section.

## 4. Extracellular Vesicles (EVs)

Extracellular vesicles (EVs) are membrane-enclosed particles released by virtually all cell types and increasingly recognized as biologically informative components of liquid biopsy. They comprise heterogeneous populations, including small EVs often enriched for exosomes, which originate from the endosomal pathway, and larger vesicles generated by outward budding of the plasma membrane. EVs contain proteins, lipids, metabolites, DNA, mRNA, long non-coding RNAs, circular RNAs, and miRNAs, reflecting both the cell of origin and the biological state of the releasing cell. In cancer, EVs participate in intercellular communication, tumor–stromal crosstalk, immune modulation, angiogenesis, epithelial–mesenchymal transition, and metastatic niche formation. Their abundance in biofluids, lipid bilayer-mediated protection of molecular cargo, and capacity to carry tumor-enriched signals make them attractive substrates for liquid biopsy [27].

In thyroid cancer, EV-associated miRNAs have received particular attention. Unlike bulk circulating miRNAs, EV-associated miRNAs are protected from RNase-mediated degradation and may reflect active, regulated cargo loading rather than passive release from dying cells. This distinction is biologically important because miRNA sorting into EVs is not random. Specific sequence motifs and RNA-binding proteins can promote preferential export of selected miRNAs into small EVs, whereas other miRNAs are retained within the cell [28]. Accordingly, EV-associated miRNAs may represent an actively secreted molecular output of tumor and stromal cells, rather than simply a cleaner version of total circulating miRNAs. This provides a strong rationale for evaluating EV-miRNAs separately from free serum/plasma miRNAs.

**Figure 4:**
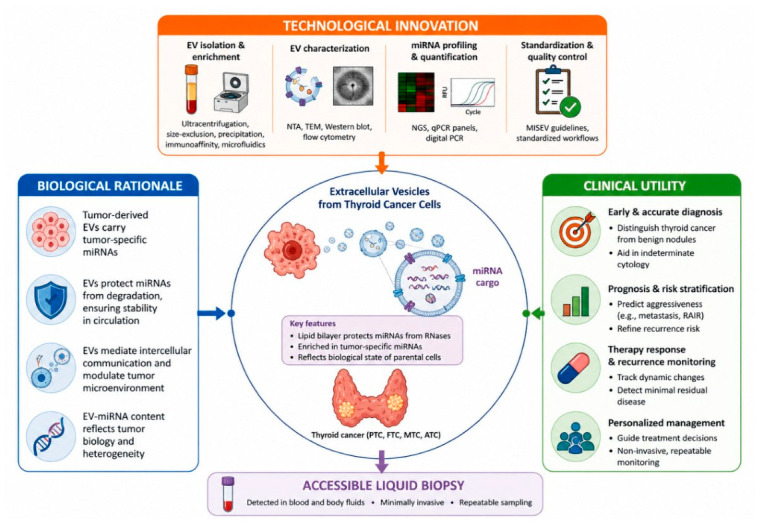
Extracellular vesicle-associated miRNAs in thyroid cancer. Schematic representation of the biological origin, technological approaches for isolation and profiling, and clinical applications of circulating EV-derived miRNAs, highlighting their role as stable, tumor-enriched biomarkers for diagnosis, risk stratification, and disease monitoring in thyroid cancer.

The analytical advantages of EVs are counterbalanced by substantial methodological challenges [29]. EV preparations may vary according to blood collection tubes, processing time, centrifugation protocols, storage conditions, and isolation methods. Ultracentrifugation, density-gradient separation, size-exclusion chromatography, precipitation-based kits, immunoaffinity capture, and microfluidic platforms differ in yield, purity, scalability, and risk of co-isolating non-vesicular RNA–protein or lipoprotein complexes. Therefore, EV studies require careful characterization of vesicle size, concentration, morphology, and marker expression, as well as assessment of potential non-vesicular contaminants. The Minimal Information for Studies of Extracellular Vesicles 2023 (MISEV2023) consensus guidelines provide a standardized framework recommending the use of complementary methods, such as nanoparticle tracking analysis, transmission electron microscopy, and immunodetection of EV-enriched and EV-depleted markers [29,30]. Without such controls, differences attributed to EV cargo may partly reflect technical variation or contamination. Figure 4 provides an integrated overview of EV-associated miRNAs in thyroid cancer.

Early integrative reviews emphasized both the promise and limitations of EV-based biomarkers in thyroid cancer, particularly the heterogeneity of EV isolation methods and downstream miRNA quantification [30]. A systematic review and meta-analysis published in 2021 suggested that EV-associated miRNAs could discriminate malignant from benign thyroid nodules with pooled sensitivities and specificities exceeding 75%, especially when multi-miRNA panels were used [31]. Recurrent signals included miR-146b-5p, miR-221/222, and miR-182, supporting biological plausibility. However, the included studies were generally small, geographically concentrated, and methodologically heterogeneous, limiting definitive clinical conclusions.

The most recent original studies are summarized in Table 2. Three European studies—Delcorte et al., Capriglione et al., and D’Amico et al.—share a broadly similar translational objective: to determine whether circulating EV-associated miRNAs can discriminate PTC from benign thyroid disease using plasma or serum samples [32,33,34]. Importantly, these studies consistently suggest that EV-miRNA profiling may reveal tumor-associated signals that are weak or absent in unfractionated plasma or serum.

Delcorte et al. used a highly controlled paired tissue–plasma–EV design, combining density cushion and size-exclusion chromatography with extensive EV characterization [33]. Candidate miRNAs were not discriminatory in whole plasma, whereas EV-associated miR-146b-5p and miR-21-5p significantly differentiated PTC from benign goiter. EV particle counts did not differ between groups, suggesting that cargo composition rather than total EV abundance carried the diagnostic signal. This design provides strong support for EV-specific enrichment, although the study was limited by modest sample size and a narrow biomarker panel. In addition, miRNA levels did not change 12–14 days after thyroidectomy, which challenges the assumption of a tumor-derived origin and contrasts with studies demonstrating postoperative normalization of EV-associated miRNAs.

Capriglione et al. adopted a broader discovery-driven strategy, using polymer-based EV isolation from serum, TaqMan miRNA arrays, and qRT-PCR validation in an independent cohort [32]. The authors identified a four-miRNA EV signature that discriminated PTC from controls. They also directly compared EV-associated and free serum miRNAs and found limited correlation between compartments, supporting selective miRNA packaging into EVs. However, precipitation-based EV isolation may co-isolate non-vesicular material, and associations with nodal metastasis were inconsistent across cohorts, limiting conclusions regarding prognostic utility.

D’Amico et al. added a longitudinal perioperative perspective by evaluating EV-miRNAs before and after thyroidectomy in patients with PTC and benign goiter [34]. The study demonstrated that selected EV-miRNAs were elevated in PTC and decreased after surgery, supporting a tumor-related origin and potential value for disease monitoring. Nevertheless, this was a proof-of-concept study with a small cohort, short postoperative follow-up, and a restricted miRNA panel, and was not powered for formal diagnostic accuracy assessment.

Together, these European studies converge on the conclusion that EV-associated miRNAs may capture clinically relevant tumor-derived signals not reliably detectable in whole plasma or serum. At the same time, they illustrate how differences in EV purification, miRNA selection strategy, postoperative sampling, and cohort design can substantially influence results. Their collective limitations reinforce the need for harmonized EV workflows and larger prospective validation studies.

A second major line of evidence comes from three studies by Li G et al., from the same research group in China, which progressively extend EV-miRNA analysis from diagnostic discrimination to clinically relevant phenotypes such as radioiodine refractoriness, metastatic behavior, and follicular-patterned malignancy [35,36,37]. In a translational study comparing RAIR and radioiodine-avid PTC, small RNA sequencing identified EV-associated miR-1296-5p as selectively enriched in RAIR models and patient plasma EVs [35]. This miRNA showed high diagnostic accuracy for RAIR status and emerged as an independent clinical risk factor. Mechanistic experiments linked miR-1296-5p to repression of the sodium–iodide symporter, providing biological coherence with radioiodine refractoriness. Clinically, this work suggests that EV-miRNAs may help non-invasively identify treatment-refractory disease, a setting in which conventional biomarkers are often insufficient.

In a subsequent study, plasma EVs from patients with metastatic and non-metastatic PTC were analyzed using miRNA microarray screening followed by TaqMan stem-loop qRT-PCR validation [36]. EV-associated miR-519e-5p was enriched in metastatic PTC, and paired analyses demonstrated that this signal was preferentially detected in the EV fraction rather than whole plasma. Although mechanistic experiments suggested roles in tumor progression and immune modulation, the principal clinical implication was the identification of an EV-miRNA marker associated with metastatic disease in patient-derived samples.

The most advanced translational application to date is the development of EV-based miRNA classifiers for follicular-patterned thyroid nodules [37]. In a multicenter diagnostic study including discovery and validation cohorts of patients with follicular thyroid carcinoma and benign follicular adenoma, plasma small EVs were isolated and profiled by high-throughput RNA sequencing. EV-derived miRNAs and long RNAs outperformed cell-free miRNAs in distinguishing malignant from benign disease. A five-miRNA EV classifier was subsequently constructed and validated by qRT-PCR, achieving high diagnostic accuracy in both training and external validation cohorts. This study directly addresses a major clinical gap, since cytology and conventional molecular testing remain imperfect for follicular-patterned lesions. Although longer follow-up and prognostic validation are still needed, the multicenter design and external validation represent an important step toward clinical implementation.

Collectively, EV-associated miRNA studies in thyroid cancer show a clear evolution from exploratory biomarker discovery toward more clinically structured translational research. Several principles emerge. First, EV fractionation can enhance signal specificity compared with bulk serum/plasma analysis. Second, EV-miRNAs appear relevant not only for diagnosis but also for identifying aggressive biological states, including metastatic PTC and RAIR disease. Third, study designs incorporating independent validation cohorts, perioperative sampling, and multicenter recruitment provide stronger evidence than isolated case–control analyses. Remaining barriers include lack of standardized EV isolation protocols, limited comparability across platforms, small sample sizes in several studies, and the absence of prospective interventional trials demonstrating that EV-miRNA testing changes clinical management. Nevertheless, EV-associated miRNAs currently represent one of the most biologically coherent and clinically promising liquid biopsy strategies in thyroid cancer, particularly when positioned as a dedicated analyte rather than embedded within the broader and more heterogeneous category of circulating miRNAs.

## 5. Circulating Tumor Cells

**Figure 5:**
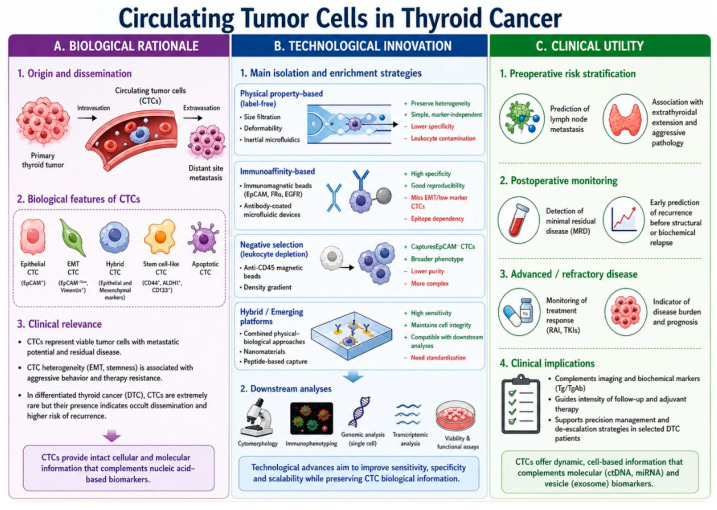
Schematic representation of circulating tumor cells (CTCs) in thyroid cancer, illustrating their release into the bloodstream, isolation technologies, and clinical applications in diagnosis, prognosis, and treatment monitoring.

Circulating tumor cells (CTCs) represent a biologically compelling component of liquid biopsy, reflecting the cellular intermediates of metastatic dissemination and tumor persistence. An integrated overview of CTCs in thyroid cancer can be found in Figure 5. Unlike circulating tumor DNA or EVs, CTCs retain intact cellular structure and phenotype, enabling direct interrogation of tumor heterogeneity, epithelial–mesenchymal plasticity, and stemness features at the single-cell level. In solid tumors, CTCs have demonstrated prognostic and predictive relevance, particularly in advanced disease. In thyroid cancer, however, their clinical translation has been slower, largely due to the typically indolent biology of differentiated tumors and the consequent rarity of detectable CTCs in peripheral blood. Systematic evidence synthesized up to 2022 indicates that, despite low abundance, CTCs detection in PTC is associated with recurrence risk, while in MTC it correlates with metastatic burden and survival, positioning CTCs primarily as prognostic and monitoring biomarkers rather than diagnostic tools [38,39].

Beyond their biological relevance, the clinical translation of circulating tumor cells critically depends on the ability to reliably isolate and characterize these extremely rare cells from peripheral blood. Over the past decade, a wide spectrum of technologies has been developed to enrich and detect CTCs by exploiting either their physical properties or their biological features, each with distinct strengths and limitations (Figure 5) [3,4]. Size- and deformability-based approaches, including filtration and microfluidic inertial focusing, enable label-free capture of CTCs and preserve phenotypic heterogeneity, but suffer from limited specificity and potential leukocyte contamination. Immunoaffinity-based methods, most commonly targeting epithelial markers such as EpCAM through immunomagnetic enrichment or microfluidic antibody-coated devices, offer high specificity and analytical reproducibility, yet may systematically miss CTCs subpopulations that have undergone epithelial–mesenchymal transition or display low marker expression. Negative selection strategies that deplete leukocytes (e.g., CD45-based subtraction) partially overcome this limitation but often yield lower purity. More recent platforms integrate nanomaterials, peptide-based capture, or hybrid physical–biological principles to improve sensitivity while maintaining cell integrity, enabling downstream cytology, immunophenotyping, and molecular analyses at the single-cell level. Despite substantial technological advances, CTCs detection in thyroid cancer remains challenged by extreme cellular rarity, biological heterogeneity, and lack of assay standardization, underscoring the need for harmonized methodologies and prospective validation before widespread clinical implementation. Against this backdrop, recent original studies have progressively shifted the field from proof-of-concept enumeration toward biologically informed detection strategies and clinically actionable applications (see Table 3).

An early demonstration of the dynamic nature of CTCs in differentiated thyroid cancer came from a prospective paired-sample study evaluating CTCs levels before and after radioiodine therapy (Table 3) [40]. Using peripheral blood mononuclear cell isolation followed by immunofluorescent identification of CD45-negative, EpCAM-positive cells, the investigators showed a significant reduction in CTCs positivity and absolute counts following radioiodine administration. This study was conceptually important in establishing CTCs as a potential pharmacodynamic marker reflecting therapeutic response rather than a static prognostic indicator. However, the reliance on EpCAM-based capture likely underestimated EMT-shifted CTCs, baseline counts were low, and follow-up was insufficient to link early CTCs dynamics to long-term outcomes such as recurrence or survival.

In 2022, two complementary studies expanded the biological and clinical framing of CTCs in thyroid cancer. One proposed a “CTCs-based cytology” approach for assessing minimal residual disease in PTC [41]. By combining rare-cell enrichment with cytomorphologic and immunocytologic evaluation, this work aimed to approximate the diagnostic logic of tissue cytology in a blood-based format, particularly in scenarios where conventional imaging and biochemical markers are inconclusive. While feasibility was demonstrated, the study underscored challenges intrinsic to cytology-based liquid biopsy, including pre-analytical variability, the risk of benign epithelial contamination, and the absence of integrated molecular confirmation.

In parallel, a biologically oriented investigation focused on epithelial–mesenchymal transition (EMT) and stemness features of CTCs, incorporating phenotypic classification and CD133 expression as markers of aggressive disease biology [42]. This observational study showed that EMT-like and CD133-positive CTCs were associated with worse clinicopathologic features and inferior survival, suggesting that CTCs phenotype may convey more prognostic information than enumeration alone. These findings aligned with broader oncologic evidence that mesenchymal and stem-like CTCs subsets disproportionately contribute to metastatic competence. Limitations included single-timepoint sampling, lack of standardized EMT thresholds, and restricted generalizability due to modest cohort size.

A major advance in the field occurred with the introduction of large-scale, preoperative CTCs studies in PTC. In 2024, a retrospective cohort of 1478 patients undergoing evaluation for thyroid nodules assessed folate receptor–positive CTCs (FR + CTCs) using immunomagnetic leukocyte depletion followed by folate receptor–targeted oligonucleotide labeling and quantitative PCR readout [43]. By including both malignant and benign nodules, this study enabled evaluation of diagnostic performance under real-world prevalence conditions. FR + CTCs levels were higher in malignant cases and correlated with adverse clinicopathologic features, including tumor size and nodal involvement. However, diagnostic accuracy as a standalone test was modest, reinforcing the notion that CTCs should complement rather than replace ultrasound and cytology. The retrospective design, potential confounding factors influencing folate receptor assays, and lack of prospective decision-impact analysis limited immediate clinical translation.

Also in 2024, a prospective longitudinal study examined postoperative CTCs kinetics following thyroidectomy in PTC [44]. Serial sampling demonstrated that persistent postoperative CTCs positivity was associated with higher recurrence risk, supporting the concept of CTCs as markers of minimal residual disease. This design directly addressed a clinically relevant question—whether early postoperative blood-based signals can refine dynamic risk stratification beyond static pathology. Nevertheless, follow-up duration was limited, and perioperative inflammatory effects on CTCs detection could not be fully excluded.

A separate 2024 study focused on metastatic papillary thyroid microcarcinoma, a subgroup that challenges traditional size-based risk stratification paradigms [45]. Preoperative CTCs assessment identified a subset of microcarcinomas with systemic dissemination potential, suggesting that CTCs may capture aggressive biology not apparent from primary tumor dimensions alone. While conceptually attractive for guiding de-escalation versus definitive intervention, this retrospective analysis was subject to selection bias and requires validation in broader, prospectively followed cohorts.

The 2025 studies further refined the clinical niche of CTCs by concentrating on locoregional aggressiveness in PTC. One investigation restricted analysis to unifocal PTC to minimize biological heterogeneity and demonstrated that preoperative CTCs positivity was independently associated with lymph node metastasis [46]. This finding is clinically relevant, as unifocal tumors are often considered lower risk, yet a subset harbors occult nodal disease. Another contemporaneous study linked elevated CTCs levels to capsular invasion and nodal spread, reinforcing the association between circulating tumor burden and invasive behavior [47]. Across both studies, the principal limitation was the retrospective surgical-series design, with potential confounding from institutional practices regarding lymph node dissection and patient selection.

The most recent contribution represents a technological and conceptual advance in CTCs capture and risk stratification. A prospective study in approximately 200 patients with PTC introduced a peptide–magnetic nanoparticle platform (“Tumorfisher”) designed to enhance capture efficiency for rare epithelial CTCs [48]. Analytical validation using spike-in experiments demonstrated EpCAM-dependent capture efficiency, followed by immunofluorescent identification of DAPI-positive, cytokeratin-positive, CD45-negative cells. Clinically, the study proposed a dual-threshold model, with higher cutoffs serving as high-specificity rule-in markers for aggressive disease and lower cutoffs offering high negative predictive value for indolent tumors. Importantly, CTCs-negative patients exhibited superior progression-free survival over a median follow-up of nearly four years, particularly within the papillary thyroid microcarcinoma subgroup. These findings suggest that optimized CTCs platforms may operationalize biologically informed de-escalation strategies. However, limited representation of distant metastatic cases and continued reliance on EpCAM expression underscore the need for broader validation and EMT-inclusive capture approaches.

In summary, recent advances in circulating tumor cell research in thyroid cancer reflect a clear evolution from exploratory enumeration toward biologically grounded, clinically contextualized applications. Systematic evidence establishes CTCs as prognostic indicators in both PTC and MTC, while original studies published since 2021 increasingly embed CTCs assessment into clinically meaningful decision points, including preoperative risk enrichment, postoperative surveillance, and treatment response monitoring. Technological innovations—ranging from receptor-based molecular assays to peptide–nanoparticle capture platforms—have improved sensitivity and scalability, partially overcoming the challenge of low CTCs abundance in indolent disease. Nevertheless, substantial hurdles remain, including methodological heterogeneity, lack of standardized cutoffs, limited longitudinal outcome data, and uncertain incremental value over optimized conventional risk models. Taken together, the current body of evidence supports CTCs as a promising adjunct biomarker in selected thyroid cancer settings, while underscoring the need for harmonized platforms and prospective, outcome-driven trials to define their precise role within contemporary, de-escalation-oriented thyroid cancer management.

## 6. Conclusions and Future Directions

Liquid biopsy in thyroid cancer is transitioning from analytical feasibility to clinical decision relevance. This maturation is particularly important in a disease spectrum where many differentiated thyroid cancers (DTC) are low-shedding and clinically indolent, while poorly differentiated/anaplastic thyroid cancer (PDTC/ATC) and advanced metastatic disease provide a clearer window in which circulating biomarkers can meaningfully inform real-time management. Accordingly, the central question is no longer “can we detect a signal?”, but rather “in which scenario does a given analyte change clinical decisions beyond imaging, cytology, and conventional biomarkers?”

Across analytes, thyroid cancer illustrates a strong biology–technology interdependence. ctDNA is most compelling for advanced/RAIR DTC, PDTC/ATC, and MTC, where higher tumor fraction and rapid kinetics enable treatment monitoring, early molecular progression signals, and identification of actionable alterations (including resistance mechanisms). In contrast, EV cargo—particularly EV-miRNAs—appears comparatively well-suited for diagnostic and phenotypic stratification questions where free-circulating RNA is diluted by non-tumor background. Circulating miRNA studies further emphasize that “bulk” serum/plasma and EV-enriched fractions are not analytically interchangeable, and that fraction-resolved designs may be essential for reproducibility. CTCs remain biologically attractive due to intact cellular phenotype, but their clinical translation in thyroid cancer will likely depend on platform harmonization, phenotype-inclusive capture (beyond purely epithelial markers), and outcome-driven validation demonstrating incremental value over optimized clinicopathologic risk models.

A consistent barrier across the liquid biopsy landscape is standardization: pre-analytical variables (tube type, processing time, storage), isolation methods (particularly for EVs), assay chemistry, bioinformatic pipelines, and calling thresholds can dominate between-study variability and inflate apparent performance. One of the main limitations shared by conventional isolation methods used for liquid biopsy analytes is their inability to selectively isolate targets without co-enriching other blood components, resulting in suboptimal purity. This highlights the need for the development of advanced technologies capable of overcoming these constraints. In this context, microfluidic-based approaches have emerged as a promising alternative. These technologies have already been implemented in the study of highly prevalent and aggressive cancers. However, their application in thyroid cancer remains limited, highlighting a significant opportunity for their integration as a valuable tool in this field.

In addition, interpretative frameworks must explicitly address confounders such as clonal hematopoiesis for cfDNA-based assays. For relevant translational impact, future studies should increasingly move from retrospective case–control designs toward prospective, multicenter cohorts with prespecified intended use (rule-in vs rule-out; baseline genotyping vs. longitudinal monitoring; MRD vs. progression), clinically meaningful endpoints, and decision-impact metrics. Looking forward, several key emerging themes are poised to reshape thyroid cancer liquid biopsy. First, cfDNA analysis is moving beyond mutation-centric testing toward multi-signal approaches—methylation profiling and fragmentomics—that may improve sensitivity and tissue-of-origin inference in low-shedding contexts [49,50]. Second, minimal residual disease (MRD) is evolving from a prognostic concept to an actionable endpoint, motivating tumor-informed designs and prospective intervention trials where molecular relapse triggers escalation or de-escalation [8,51]. Third, regulatory science and community standards are becoming central, with increasing emphasis on reference materials, analytical validation, and harmonized reporting frameworks [52,53]. Fourth, EV research is advancing toward higher-resolution readouts (including single-EV analytics) and computationally assisted discovery (AI/ML feature selection and multi-omics integration), which may help overcome biological heterogeneity and signal dilution [54,55]. Finally, multi-cancer early detection paradigms highlight the need to quantify downstream diagnostic burden, overdiagnosis risk, cost-effectiveness, and equity of access [56]. These issues are especially relevant to thyroid cancer because nodules are so often found incidentally, and the current medical approach favors de-escalation-oriented management and avoiding over-treatment.

In summary, the most credible path to implementation is an outcomes-driven framework: define a narrow clinical question (rule-in vs. rule-out diagnosis; MRD vs. progression; resistance detection vs. target discovery), match it to the analyte with the strongest biological fit (e.g., EV-miRNA for diagnostic enrichment; tumor-informed ctDNA for MRD in high-risk settings; ctDNA and cfRNA for the detection of actionable alterations, including gene fusions), and validate within prospective, multicenter designs using standardized workflows and decision-impact endpoints. Ultimately, multi-analyte integration—combining ctDNA/cfRNA, EV cargo, and CTCs phenotyping—may offer the best balance between sensitivity and interpretability in a disease where single-marker approaches often underperform. In that sense, thyroid cancer represents both a challenge and an opportunity: a paradigm in which the value of liquid biopsy will be defined not by detectability alone, but by its ability to refine de-escalation, personalize systemic therapy, and support truly dynamic disease management.

## Figures and Tables

**Table 1:** Recent original ctDNA studies in thyroid cancer.

Study (Author, Year)	Study Design & Population	Thyroid Cancer Type(s)	ctDNA Methodology & Technology	Main Clinical Findings	Key Limitations
Ciampi et al. 2022 [12]	Prospective peri-operative cohort; *n* = 29 sporadic cases; paired pre- and post-operative sampling (median follow-up ~8 months)	Medullary thyroid carcinoma (MTC)	Tumor-informed targeted NGS; strict preanalytics (EDTA, rapid processing); deep sequencing (~20,000× ctDNA); positivity VAF > 0.4%	Low pre-op ctDNA detection (15.4%) but enrichment in aggressive disease (RET M918T, higher tumor VAF); post-op ctDNA positivity associated with elevated calcitonin/CEA and persistent disease	Small cohort; relatively high VAF cutoff limits low-level MRD detection; heterogeneous post-op sampling times; CH not studied
Tarasova et al. 2024 [13]	Large retrospective database analysis; *n* = 1094 plasma samples (2016–2021)	Mixed; enriched for advanced disease (ATC, PTC, FTC, MTC, PDTC)	Tumor-agnostic commercial plasma NGS panel (Guardant360); bTMB calculated in subset; CH assessed.	≥1 alteration detected in 78.3%; subtype-specific landscapes (e.g., BRAF V600E in PTC/ATC, RAS in FTC, RET in MTC); higher bTMB in ATC	No systematic tissue concordance; limited staging/therapy annotation; hypothesis-generating rather than outcome-defining
Thomaz et al. 2025 [19]	Cross-sectional feasibility study; *n* = 34; correlation with ATA response categories	Differentiated thyroid cancer (mixed)	Tumor-informed digital PCR for ctDNA and cfRNA (including fusions)	ctDNA/ctRNA detected in all structural incomplete responses; 91% of excellent responses ctDNA-negative; cfRNA fusion detected despite undetectable thyroglobulin in one case	Small sample size; cross-sectional design; limited longitudinal outcome data; CH not studied
Hamidi et al. 2025 [20]	Retrospective cohort; *n* = 45 baseline; longitudinal monitoring in *n* = 31 (130 samples)	Anaplastic thyroid carcinoma (ATC)	Plasma ctDNA monitoring (NGS-based); baseline and serial assessments	ctDNA concordant with disease status in 93%; surveillance sensitivity ~77–78% with 100% specificity/PPV for recurrence/progression; false negatives linked to low burden or restricted metastatic sites	Retrospective design; false negatives in low-shedding scenarios; molecular heterogeneity; CH not studied
Wijewardene et al. 2025 [21]	Prospective cohort (2020–2024); *n* = 40	Advanced/metastatic MTC, PDTC, ATC	Plasma cfDNA NGS (50-gene panel; Ion Torrent Genexus); Streck tubes; serial sampling	cfDNA mutations detected in 50% overall; higher sensitivity pre-TKI (86%) vs on-therapy (54%); higher cfDNA levels and rising cfDNA associated with worse PFS	Limited panel (e.g., no TERT promoter, limited fusion detection); small subtype-specific numbers; reduced shedding under effective therapy; CH not studied

**Table 2:** Summary of original studies on circulating EV-derived miRNAs in thyroid cancer.

Study (Author, Year)	Study Design & Population	Thyroid Cancer Type(s)	EV Isolation/miRNA Detection Methodology	Main Clinical Findings	Key Limitations
Delcorte et al., 2022 [33]	Case–control, paired tissue–plasma–EV analysis; *n* ~40	PTC vs. benign nodules	Iodixanol density cushion and size exclusion chromatography/Targeted qRT-PCR (TaqMan assays) on EV-RNA	In purified plasma-derived EVs, only miR-146b-5p and miR-21-5p among six candidates were significantly different between PTC and benign thyroid disease. No discriminatory signal was observed in bulk plasma, supporting EV-specific enrichment.	Small sample size, limited miRNA panel, miRNA levels did not change after thyroidectomy
Capriglione et al., 2022 [32]	Two-stage study: discovery + independent validation; *n* ~90	PTC, benign nodules, healthy controls	Polymer-based EV isolation from serum/TaqMan miRNA array → qRT-PCR validation	A four-miRNA EV signature—miR-24-3p, miR-146a-5p, miR-181a-5p, and miR-382-5p—discriminated PTC from healthy controls. Whole plasma/serum did not show comparable discrimination, and the association with nodal metastasis was inconsistent.	Small sample size, Contamination with non-vesicular material.
Li G et al., 2022 [35]	Translational study: RAIR models + clinical validation; *n* ~60	RAIR vs. RAI-avid PTC	Ultracentrifugation/Small RNA sequencing → qRT-PCR validation	EV miR-1296-5p elevated in RAIR PTC; high diagnostic accuracy; associated with NIS loss	Small sample size, unresolved function and mechanism of exosomal miR-1269-5p.
D’Amico et al., 2024 [34]	Proof-of-concept longitudinal perioperative study; *n* ~30	PTC vs. benign goiter	Ultracentrifugation/Targeted qRT-PCR on EV-miRNAs	Three EV-miRNAs—miR-1-3p, miR-206, and miR-221-3p—were elevated in PTC compared with benign disease and normalized after surgery, supporting tumor origin and potential value for postoperative monitoring.	Larger cohort, limited miRNA panel, short follow-up after surgery.
Li G et al., 2024 (JCEM) [36]	Translational study: discovery + validation cohorts; *n* > 100	metastatic vs. non-metastatic PTC	Ultracentrifugation/miRNA microarray → TaqMan stem-loop qRT-PCR	EV miR-519e-5p enriched in metastatic PTC; EV-specific discrimination of metastatic disease	Limited sample size during the initial screening phase and insufficient exploration of clinical heterogeneity.
Li G et al., 2024 (Br J Cancer) [37]	Multicenter diagnostic study: training + external validation; *n* ~190	FTC vs. benign follicular adenomas	Ultracentrifugation/Small RNA sequencing → qRT-PCR-based EV-miRNA classifier	Five-miRNA EV classifier (miR- 127-3p, miR-223-5p, miR-432-5p, miR-146a-5p and miR-151a-3p) accurately discriminated FTC from benign nodules; EV-miRNAs outperformed cell-free miRNAs	Limited follow-up, lack of prognostic validation, need for technical validation with advanced detection technology

Abbreviations: EV, extracellular vesicle; PTC, papillary thyroid carcinoma; FTC, follicular thyroid carcinoma; RAIR, radioiodine-refractory; RAI, radioactive iodine; qRT-PCR, quantitative reverse transcription PCR; NIS, sodium–iodide symporter.

**Table 3:** Summary of Original Studies on Circulating Tumor Cells (CTCs) in Thyroid Cancer.

Study (Author, Year)	Study Design & Population	Thyroid Cancer Type(s)	CTCs Methodology & Technology	Main Clinical Findings	Key Limitations
Schmidt, 2021 [40]	Prospective paired study; *n* ~55	DTC, before and after radioidine therapy	EpCAM-based immunofluorescence CTCs detection	CTCs counts decreased after radioiodine therapy	Low counts; EpCAM dependence; no long-term outcomes
Innaro, 2022 [41]	Observational cohort; *n* ~40	PTC after initial therapy	CTCs enrichment with cytology-like evaluation	Feasible cytological assessment from blood	Small cohort; lack of molecular confirmation
Li D, 2022 [42]	Retrospective prognostic cohort; *n* ~349	PTC, FTC, MTC, ATC	EMT and CD133 phenotyping of CTCs	EMT+/CD133+ CTCs associated with poor prognosis	Single time-point; EMT marker specificity
Zeng Q, 2024 [43]	Retrospective preoperative cohort; *n* ~1478	Malignant group vs. Benign group	FR + CTCs by immunomagnetic depletion + qPCR	Higher FR + CTCs in malignant and aggressive tumors	Retrospective; moderate diagnostic accuracy
Yu HW, 2024 [44]	Prospective postoperative PTC cohort; *n* ~62	PTC before and after 2 weeks and 3 months	FR + CTCs longitudinal monitoring	Persistent CTCs linked to recurrence	Short follow-up; perioperative confounding
Yu M, 2024 [45]	Retrospective cohort; *n* ~705	PTC and Lymph node metastasis	FR + CTCs preoperative detection	CTCs identify aggressive microcarcinomas	Selection bias; retrospective design
Gu Y, 2025 [46]	Retrospective cohort; *n* ~507	PTC and Lymph node metastasis	Preoperative FR + CTCs assessment	CTCs positivity predicts nodal disease	Single-center; surgical bias
Yu M, 2025 [47]	Retrospective cohort; *n* ~746	PTC and Capsular invasion and nodal spread	FR + CTCs correlated with pathology	CTCs associated with invasive features	Incremental value not fully assessed
Jiang L, 2026 [48]	Prospective technology-driven cohort; *n* ~200	Preoperative PTC	Tumorfisher peptide–nanoparticle CTCs capture	Dual-threshold model predicts recurrence risk	Limited metastatic cases; EpCAM reliance

## Data Availability

No new data were created or analyzed in this study.
